# Pharmacological thromboprophylaxis as a risk factor for early periprosthetic joint infection following primary total joint arthroplasty

**DOI:** 10.1038/s41598-022-14749-y

**Published:** 2022-06-22

**Authors:** Fu-Yuan Pai, Wei-Lin Chang, Shang-Wen Tsai, Cheng-Fong Chen, Po-Kuei Wu, Wei-Ming Chen

**Affiliations:** 1 Department of Orthopaedics and Traumatology, Taipei Veterans General Hospital, No. 201, Sec 2, Shi-Pai Road, Taipei, 112 Taiwan; 2 Department of Orthopaedics, School of Medicine, National Yang Ming Chiao Tung University, Taipei, Taiwan

**Keywords:** Medical research, Outcomes research

## Abstract

Venous thromboembolism (VTE) prophylaxis has been suggested for patients who underwent total join arthroplasty (TJA). However, the morbidity of surgical site complications (SSC) and periprosthetic joint infection (PJI) has not been well evaluated. We aimed to evaluate the impact of VTE prophylaxis on the risk of early postoperative SSC and PJI in a Taiwanese population. We retrospectively reviewed 7511 patients who underwent primary TJA performed by a single surgeon from 2010 through 2019. We evaluated the rates of SSC and PJI in the early postoperative period (30-day, 90-day) as well as 1-year reoperations. Multivariate regression analysis was used to identify possible risk factors associated with SSC and PJI, including age, sex, WHO classification of weight status, smoking, diabetes mellitus (DM), rheumatoid arthritis(RA), Charlson comorbidity index (CCI), history of VTE, presence of varicose veins, total knee or hip arthroplasty procedure, unilateral or bilateral procedure, or receiving VTE prophylaxis or blood transfusion. The overall 90-day rates of SSC and PJI were 1.1% (N = 80) and 0.2% (N = 16). VTE prophylaxis was a risk factor for 90-day readmission for SSC (aOR: 1.753, 95% CI 1.081–2.842), 90-day readmission for PJI (aOR: 3.267, 95% CI 1.026–10.402) and all 90-day PJI events (aOR: 3.222, 95% CI 1.200–8.656). Other risk factors included DM, underweight, obesity, bilateral TJA procedure, younger age, male sex and RA. Pharmacological thromboprophylaxis appears to be a modifiable risk factor for SSC and PJI in the early postoperative period. The increased infection risk should be carefully weighed in patients who received pharmacological VTE prophylaxis.

## Introduction

Postoperative venous thromboembolism (VTE) is one of the most serious complications following total joint arthroplasty (TJA), including total knee arthroplasty (TKA) and total hip arthroplasty (THA)^1^. The symptomatic VTE rate in the early postoperative period might be as high as 4.3%^1^. Therefore, pharmacological VTE prophylaxis with warfarin, low molecular weight heparin (LMWH), factor Xa inhibitors (fondaparinux, apixaban, dabigatran, rivaroxaban), or aspirin has been suggested in several guidelines^1,2^. Because of the lower rates of VTE in Asian population^3–5^, pharmacological VTE prophylaxis would be administered for Asian patients undergoing TJA procedures based on VTE and bleeding risk^6,7^. For patients who had history of VTE, varicose veins, congestive heart failure, thromboembolic stroke, family history of VTE, underwent a simultaneous bilateral TJA procedure or a prolonged procedure (> 2 h), the VTE risk was considered elevated^6^. Factors associated with increased bleeding risk included advanced age, active cancer with or without metastasis, liver or renal failure, thrombocytopenia, hemophilia and other bleeding disorders^6^. For patients without elevated VTE risk, mechanical prophylaxis alone without the need for pharmacological prophylaxis was recommended from the Asia–Pacific VTE Consensus Group^6,7^. The risk of VTE and risk of bleeding were the two main factors used to justify the prophylaxis strategy^6^. However, other adverse events associated with pharmacological VTE prophylaxis should also be considered, such as surgical site complications (SSC) and periprosthetic joint infection (PJI), which have not been well evaluated. Pharmacological VTE prophylaxis has been shown to increase the risk of wound drainage. However, the risk of subsequent wound infection was not uniformly observed across all TJA procedures^7–9^.

There were only a few comparative studies that have evaluated the risk of SSC and PJI associated with VTE prophylaxis^10–12^. More evidence is required to evaluate the overall incidence and risk of infection associate with VTE prophylaxis. Therefore, we conducted this single-surgeon case series from a tertiary referral hospital over a 10-year study period in a Taiwanese population. The aim of this study was to evaluate the risk factors for SSC and PJI events in the early postoperative period. We hypothesized that VTE prophylaxis was a risk factor for early SSC and PJI events and thus these events should be given careful consideration when making treatment decisions.

## Materials and methods

### Data collection

We conducted this retrospective, single-surgeon case series in Taipei Veterans General Hospital (TVGH), a tertiary referral hospital in Taipei, Taiwan. The Institutional Review Board of TVGH approved this study. Because of the retrospective design of this observational study, the TVGH Institutional Review Board has approved our request to waive the documentation of the inform consent. We obtained medical records and images from TVGH Orthopaedic database between January 2010 and December 2019. Patients who had undergone unilateral or simultaneous bilateral primary TJA, including TKA or THA during this study period were included. We screened for patients that were eligible for analysis using Taiwan’s National Health Insurance procedure codes: “PCS-64169B, TKA” and “PCS-64162B, THA”. Indications for primary TKA procedures included primary or secondary knee osteoarthritis (ICD-10-CM code: M17), spontaneous osteonecrosis of the knee (SONK, ICD-10-CM code: M90.55, M90.56), rheumatoid arthritis of knee joint (RA, ICD-10-CM code: M05.76, M05.86, M06.86, M06.9). Indications for primary THA procedures for primary or secondary hip osteoarthritis (ICD-10-CM code M16), osteonecrosis of the femoral head (ONFH, ICD-10-CM code: M87.05, M87.15, M87.25, M87.35, M87.85) and RA of hip joint (ICD-10-CM code: M05.75, M05.85, M06.85, M08.45, M08.85, M08.95). We excluded patients that (1) underwent primary TJA following resection of musculoskeletal tumors; (2) underwent primary TJA for acute acetabular or proximal femur fractures; (3) were allergic to LMWH or aspirin; (4) had bleeding disorders (e.g. hemophilia); (5) had Child–Pugh class B or C cirrhosis; (6) had active or a history of knee or hip infection.

Three authors (WLC, FYP and SWT) reviewed all medical records. We recorded age, sex, height, weight, body mass index (BMI), history of smoking, diabetes mellitus (DM), rheumatoid arthritis (RA), Charlson comorbidity index (CCI), history of venous thromboembolism (VTE), presence of varicose vein, type of procedure (TKA or THA), unilateral or bilateral procedure, receipt of VTE prophylaxis or transfusion, length of stay (LOS) and in-hospital mortality.

### Protocol for thromboprophylaxis of venous thromboembolism

During January 2010 to December 2017, the indications for VTE prophylaxis included BMI ≥ 30 (kg/m^2^), presence of varicose veins, or a history of deep vein thrombosis (DVT) or pulmonary embolism (PE). However, in December 2017, a patient underwent simultaneous bilateral TKA who did not meet the criteria of VTE prophylaxis mentioned above but developed PE during the perioperative period. Therefore, simultaneous bilateral TKA or THA was added as an indication for VTE prophylaxis from January 2018.

The thromboprophylaxis protocol consisted of an injection of low molecular weight heparin (LMWH, Enoxaparin, Clexane, 2000 IU, 0.2 cc) at the post-anesthesia care unit (PACU) immediately after surgery and every day until post-operative day (POD) 3. On POD 4, LMWH was switched to low-dose aspirin (Bokey^®^, 100 mg) for 2 weeks. If the patient was diagnosed with a DVT based on clinical symptoms and positive ultrasonography, low-dose aspirin would then be given for a total of 5 weeks.

### Surgical technique and post-operative care

All procedures were performed by a single fellowship-trained orthopedic surgeon (WMC) under spinal or general anesthesia. All the TKA procedures were performed through a minimally invasive mid-vastus approach^13^. The TKA components were fixed with cement. A tourniquet and a close-suction drain were used in every TKA procedure. The types of the TKA prostheses included Nexgen (Zimmer Biomet, Warsaw, IN, USA), NRG (Stryker, Mahwah, NJ, USA) and Triathlon (Stryker, Mahwah, NJ, USA). All the THA procedures were performed through a minimally invasive trans-gluteal approach^14^. All the THA components were cementless. A close-suction drain was used in every THA procedure. The only type of THA prosthesis was the Trident cup (Stryker, Mahwah, NJ, USA) and SecurFit femoral stem (Stryker, Mahwah, NJ, USA). For patients who received a TKA procedure, a single-dose, intra-articular tranexamic acid was given after wound closure. For patients who underwent a THA procedure, a single-dose, intravenous tranexamic acid was given immediately before incision.

Intravenous prophylactic antibiotics were administered for 24 h after the TJA procedure unless there was evidence of any infection. All patients started rehabilitation and ambulation on POD 1. We routinely checked hemoglobin level on POD 1. If the hemoglobin level was < 9.0 g/dL or between 9.0–10.0 g/dL with symptoms of anemia, including malaise, dizziness, hypotension or tachycardia, we would transfuse the patients with 1–2 units of packed red blood cells.

### Outcome domains

We aimed to evaluate the incidence of SSC and PJI at different time points. SSC was defined as hematoma, seroma, delayed wound healing, or superficial wound infection that required systemic antibiotics or a surgical debridement procedure. PJI is a more severe type of infection involving the bone and prosthesis surface that required extensive debridement or removal of the prosthesis. The outcome domains included 30-day and 90-day readmission for SSC or PJI, 90-day SSC or PJI events, and 1-year reoperation for SSC or PJI. The 30-day and 90-day readmission included a hospital readmission or a return visit to the emergency department. The all 90-day SSC or PJI events included readmissions and events that were identified and treated at the outpatient follow-up visits.

### Statistical analysis

Statistical analyses for this study were performed using SPSS version 22 (IBM Corp., Armonk, NY). Descriptive statistics were performed for all available data. The Chi-square test was used for comparing discrete variable. When one or more of the cells in the contingency table had an expected frequency of less than 5, we performed the Fisher’s exact test. A multivariate logistic regression analysis was performed to determine risk factors for SSC and PJI, including age, sex, WHO classification of weight status^15^, smoking, DM, RA, CCI, history of VTE, presence of varicose veins, TKA or THA procedure, unilateral or bilateral procedure, or receiving VTE prophylaxis or blood transfusion. The backward variable selection method was employed to choose the optimal model, with the significance test for a risk factor entering and remaining at the significance level of 0.05. The results were expressed as an odds ratio with a 95% confidence interval (CI).

### Ethics approval and consent to participate

The study was approved by the ethical committee of Taipei Veterans General Hospital. Because of the retrospective design of this observational study, the TVGH Institutional Review Board has approved our request to waive the documentation of the inform consent. The study was in accordance with the Declaration of Helsinki and all procedures were performed in accordance with relevant guidelines and regulations.

## Results

### Outcome

A total of 7511 patients who had undergone a TJA procedure were included for analysis. The rates of 90-day symptomatic VTE events in patients who received pharmacological thromboprophylaxis and those did not were 0.20% (N = 4) and 0.52% (N = 29), respectively. The patient demographics have been shown in Table 1. The overall rates of 30-day readmission for SSC and PJI were 0.8% (N = 58) and 0.1% (N = 9), respectively. The overall rates of 90-day readmission for SSC and PJI were 0.9% (N = 71) and 0.2% (N = 12), respectively. The overall incidence of SSC and PJI within 90 days after the TJA procedure were 1.1% (N = 80) and 0.2% (N = 16), respectively. The overall 1-year reoperation rates for SSC and PJI were 0.1% (N = 11) and 0.4% (N = 29), respectively. The rates of SSC and PJI stratified by the administration of VTE prophylaxis are shown in Table 2. The rates of 30-day (1.1% vs. 0.6%, p = 0.041), 90-day readmission for SSC (1.4% vs. 0.8%, p = 0.011) and the overall incidence of SSC (1.5% vs. 0.9%, p = 0.039) and PJI (0.4% vs. 0.1%, p = 0.037) within 90 days after the TJA procedure were higher in patients who received pharmacological thromboprophylaxis, compared with those who did not (Table 2).

**Table 1:** Patient demographics.

	Overall (N = 7511)
Age (years)	68.7 ± 11.2 (range 17–97)
**Sex (%)**	
Female	5709 (76.0%)
Male	1802 (24.0%)
Height (cm)	155.3 ± 8.3 (range 118–195)
Weight (kg)	65.6 ± 12.1 (range 31–160)
Body mass index (kg/m^2^)	27.2 ± 4.4 (range 14.2–50.0)
**WHO classification of weight status**	
Underweight (BMI < 18.5)	102 (1.4%)
Normal weight (18.5 ≤ BMI < 25)	2316 (30.8%)
Pre-obesity (25 ≤ BMI < 30)	3356 (44.7%)
Obesity class I (30 ≤ BMI < 35)	1355 (18.0%)
Obesity class II (35 ≤ BMI < 40)	301 (4.0%)
Obesity class III (BMI ≥ 40)	81 (1.1%)
**Smoking (%)**	
Yes	623 (8.3%)
No	6888 (91.7%)
**DM (%)**	
Yes	1586 (21.1%)
No	5925 (78.9%)
**RA (%)**	
Yes	196 (2.6%)
No	7315 (97.4%)
**Charlson comorbidity index (%)**	
0	377 (5.0%)
1	508 (6.8%)
2	1483 (19.7%)
3	2271 (30.2%)
4	1655 (22.0%)
5	776 (10.3%)
6+	441 (5.9%)
**History of VTE (%)**	
Yes	16 (0.2%)
No	7495 (99.8%)
**Presence of varicose veins (%)**	
Yes	197 (2.6%)
No	7314 (97.4%)
**Type of procedure (%)**	
TKA	5486 (73.0%)
THA	2025 (27.0%)
**Unilateral vs. bilateral procedure (%)**	
Unilateral	5881 (78.3%)
Bilateral	1630 (21.7%)
**Pharmacological VTE prophylaxis (%)**	
Yes	1957 (26.1%)
No	5554 (73.9%)
Transfusion rate (%)	2629 (35.0%)
Length of stay (days)	4.7 ± 1.7 (range 2–91)
In-hospital mortality^a^ (%)	3 (0.04%)
**90-day symptomatic VTE rate (%)**	33 (0.44%)
Without pharmacological prophylaxis	29 (0.52%)
With pharmacological prophylaxis	4 (0.20%)

DM: diabetes mellitus; RA: rheumatoid arthritis; THA: total hip arthroplasty; TKA: total knee arthroplasty; VTE: venous thromboembolism.

^a^In-hospital mortality: ischemia bowel, Perforated peptic ulcer disease with sepsis, Acute myocardial infarction.

**Table 2:** Incidence of postoperative infection events.

	Overall (N = 7511)	Without pharmacological prophylaxis (N = 5554)	With pharmacological prophylaxis (N = 1957)	P-value
**30-day readmission events**				
SSC	58 (0.8%)	36 (0.6%)	22 (1.1%)	0.041
PJI	9 (0.1%)	5 (0.1%)	4 (0.2%)	0.221
**90-day readmission events**				
SSC	71 (0.9%)	43 (0.8%)	28 (1.4%)	0.011
PJI	12 (0.2%)	6 (0.1%)	6 (0.3%)	0.071
**All 90-day infection events**				
SSC	80 (1.1%)	51 (0.9%)	29 (1.5%)	0.039
PJI	16 (0.2%)	8 (0.1%)	8 (0.4%)	0.037
**1-year re-operation events**				
SSC	11 (0.1%)	7 (0.1%)	4 (0.2%)	0.440
PJI	29 (0.4%)	21 (0.4%)	8 (0.4%)	0.851

PJI: periprosthetic joint infection; SSC: surgical site complication.

### Risk factors for SSC

DM was a risk factor for 30-day (adjusted odds ratio [aOR]: 2.135; 95% CI 1.246–3.658) and 90-day (aOR: 1.673; 95% CI 1.010–2.773) readmission for SSC. The administration of pharmacological VTE prophylaxis (aOR: 1.753; 95% CI 1.081–2.842) was associated with increased risk of 90-day readmission for SSC. Obese (BMI ≥ 30 kg/m^2^) patients had an increased risk of SSC events within 90 days after the TJA procedure (aOR: 1.803; 95% CI 1.135–2.863). None of the factors recorded in this study were associated with an increased risk of 1-year reoperation for SSC (Table 3). The results of the univariate and multivariate analysis of all the registered variables were shown in Tables S1–8.

**Table 3:** Risk factors for postoperative infection events.

Independent variable	Odds ratio	95% confidence interval	P-value
**30-day readmission for SSC**			
DM	2.135	1.246–3.658	0.006
**90-day readmission for SSC**			
DM	1.673	1.010–2.773	0.046
VTE prophylaxis	1.753	1.081–2.842	0.023
**90-day SSC events**			
Obesity^a^	1.803	1.135–2.863	0.013
**30-day readmission for PJI**			
Underweight (ref: normal weight)	11.692	1.417–96.482	0.022
Bilateral procedure	4.882	1.295–18.400	0.019
**90-day readmission for PJI**			
Age	0.959	0.920–0.999	0.046
VTE prophylaxis	3.267	1.026–10.402	0.045
**90-day PJI events**			
Male sex	3.568	1.328–9.583	0.012
VTE prophylaxis	3.222	1.200–8.656	0.020
**1-year reoperation for PJI**			
Male sex	2.738	1.308–5.731	0.008
RA	5.048	1.501–16.974	0.009

DM: diabetes mellitus; PJI: periprosthetic joint infection; SSI: surgical site infection; VTE: venous thromboembolism.

^a^Including obesity class I, II and III.

### Risk factor for PJI

Underweight (BMI < 18.5 kg/m^2^, aOR: 11.692; 95% CI 1.417–96.482) and simultaneous bilateral TJA procedure (aOR: 4.882; 95% CI 1.295–18.400) were associated with an increased risk of 30-day readmission for PJI. The administration of pharmacological VTE prophylaxis was a risk factor for 90-day readmission for PJI (aOR: 3.267; 95% CI 1.026–10.402) as well as having a PJI event within postoperative 90 days (aOR: 3.222; 95% CI 1.200–8.656). Male patients were at an increased risk of 90-day PJI events (aOR: 3.568; 95% CI 1.328–9.583) and 1-year reoperation for PJI (aOR: 2.738; 95% CI 1.308–5.731). Younger age was a risk factor for 90-day readmission for PJI (aOR: 0.959; 95% CI 0.920–0.999), while RA (aOR: 5.048; 95% CI 1.501–16.974) was another risk factor for 1-year reoperation for PJI (Table 3). The results of the univariate and multivariate analysis of all the registered variables were shown in Tables S1–8.

## Discussion

The most important finding of this study was that VTE prophylaxis with a combination of LMWH and aspirin was a major risk factor for SSC and PJI in the early postoperative period. Other factors included DM, obesity, underweight, simultaneous bilateral procedures, younger age, male sex, and RA.

Risk of VTE and bleeding are two major factors to consider when choosing a thromboprophylaxis strategy^6^. However, risk of infection associated with pharmacological VTE prophylaxis was another important issue that has not been well evaluated. Pharmacological VTE prophylaxis might lead to (1) postoperative hematoma, which acts as a nidus for bacteria to settle, and (2) prolonged wound drainage, which increases wound tension, bypasses the natural barrier of the skin, increases risk of wound dehiscence and provides a retrograde pathway for pathogens^8,11,16^, but whether the pharmacological agents for prophylaxis would increase the risk of infection following TJA procedures was inconsistent^7^. The rates of SSC were generally higher in studies with routine pharmacological VTE prophylaxis than those studies in which pharmacological VTE prophylaxis were not routinely administered (0.4–13.3% vs. 1.5–3.6%)^17–20^. The rates of PJI were similar between studies with or without routine pharmacological VTE prophylaxis (0.4–1.5% vs. 0.48–1.63%)^17–20^. However, the design of these studies were case series lacked a control group. There were only a few studies that compared the risk of infection between patients who received pharmacological prophylaxis and those did not^10,11^. Parvizi et al.^11^ and Sachs et al.^10^ have concluded that the use of low-dose warfarin was associated with a higher risk of wound healing problems or infection, especially in patients who had a mean international normalized ratio greater than 1.5^11^. A meta-analysis by Hughes et al.^21^ reported similar findings, that the use of warfarin for thromboprophylaxis was associated with a higher risk of SSC and PJI, compared with aspirin. The risk of SSC and PJI were not different between the aspirin and LMWH group^21^. Runner et al.^22^ evaluated the complications of 22,072 patients who received different pharmacological agents for thromboprophylaxis. The rates of infection were higher (1.9% vs. 1.3%, p = 0.001) in patients who received more aggressive prophylaxis strategies (e.g., LMWH, warfarin, rivaroxaban, fondaparinux, or others) than the less aggressive strategies (e.g., aspirin or mechanical devices). In this study, despite the fact that a daily dose of LMWH for 3 days and then transitioning to low-dose aspirin for 2 to 5 weeks was considered a less aggressive strategy^10,11,21,22^, we still observed the association between pharmacological prophylaxis and early postoperative infection events. The postoperative 30 and 90 days were two common time points to evaluate the early postoperative complications, including SSC and PJI^23–25^. Notably, pharmacological thromboprophylaxis was associated with multiple outcome domains in the early postoperative period, including 90-day readmission for SSC, PJI, and all 90-day PJI events. To interpret these results, we might consider pharmacological thromboprophylaxis to have an impact on early postoperative infections rather than to emphasize the effect on a specific form of infection at a certain point of time (e.g., pharmacological thromboprophylaxis as a risk factor for 90-day but not 30-day readmission for PJI).

We have identified several risk factors for SSC and PJI other than VTE prophylaxis, including DM, obesity, underweight, simultaneous bilateral TJA procedure, younger age, male sex and RA. DM is associated with immune system alterations, including impaired leukocyte function, impaired release of inflammatory cytokines in response to infection stimuli^26^ and enhancing the formation of biofilm^27^. In our study, DM patients had a 1.6–2.0 times higher risk of SSC than non-DM patients, which was consistent with findings from other studies^28,29^. Notably, the risk was even higher in patients who had morning hyperglycemia^30^ and poorly controlled hemoglobin A1c values^31^, indicating that the risk of SSC in DM patients would still be modifiable. In our study, both obesity and underweight were associated with a higher risk of infection events. Obesity might lead to negative effects on the functions of immune effector cells and the expression of immunomodulatory factors^32^, poor perfusion and tissue oxygenation^33^, increased tension on wound edges and dehiscence and impaired tissue penetration of antibiotics^34^. The poor nutritional and immunocompromised status in the underweight patient might also lead to an increased risk of PJI^35^. The relationship between BMI and risk of infection was not linear; either a high or a low BMI might be associated with increased infection risk^35^. We have noted simultaneous bilateral TJA to be a risk factor for PJI, which was consistent with several large-scale studies^36,37^. However, regarding the risk of infection events, the choice between a staged or simultaneous bilateral TJA procedure remains controversial^38,39^. Younger age was another risk factor for PJI, which is consistent with the findings from several large-scale studies^40,41^. Higher activity level, greater number of loading cycles on the implant and increased wear, and prior surgery before TJA might increase the infection risk^41,42^. We found an association between male sex and PJI, which was also observed in several other studies, but the underlying mechanisms remain unclear^17,19^. The poor nutritional and immunocompromised status of RA patients might lead to an increased risk of PJI^18,40^, which was consistent with the results of this study. Because of the more liberal criteria for blood transfusion in our study, the overall transfusion rate was high (35.0%). Allogeneic blood transfusion has been reported to be associated with SSC or PJI, possibly due to transfusion-induced immunomodulation^43^. However, blood transfusion was not a risk factor for SSC or PJI in the univariate or multivariate regression analysis (Tables S1–8).

There were several limitations of this study. First, the study period was 10 years, possible modifications of the perioperative care protocol and surgical techniques could have an impact on the rates of SSI and PJI events. Second, compared with several large-scale studies^17–20^, our rates of SSC (1.1%, N = 80) and PJI (0.2%, N = 16) in the early postoperative period were low. Despite the relatively large sample size of this study (N = 7511), the number of SSC and PJI events were small. Third, we evaluated that thromboprophylaxis with a combination of LMWH and low-dose aspirin might be associated with early postoperative SSC and PJI events, but the results of this study might not be generalizable to other medications for thromboprophylaxis. Fourth, this single-surgeon study design could impact the generalizability of the results. Fifth, we should recognize the inherent limitations of the retrospective study design.

## Conclusion

Pharmacological thromboprophylaxis appears to be a modifiable risk factor for SSC and PJI in the early postoperative period. In addition to the risk of VTE and bleeding, the increased infection risk should also be carefully evaluated in patients who receive pharmacological VTE prophylaxis.

## Supplementary Information

Supplementary Table S1.

Supplementary Table S2.

Supplementary Table S3.

Supplementary Table S4.

Supplementary Table S5.

Supplementary Table S6.

Supplementary Table S7.

Supplementary Table S8.

## Data Availability

All data generated or analyzed during this study are included in this published article and available upon request.

## Abbreviations

BMI: Body mass index
CCI: Charlson comorbidity index
CI: Confidence interval
DM: Diabetes mellitus
DVT: Deep vein thrombosis
LMWH: Low molecular weight heparin
LOS: Length of stay
ONFH: Osteonecrosis of the femoral head
PACU: Post-anesthesia care unit
PE: Pulmonary embolism
PJI: Periprosthetic joint infection
POD: Post-operative day
RA: Rheumatoid arthritis
SONK: Spontaneous osteonecrosis of the knee
SSC: Surgical site complications
THA: Total hip arthroplasty
TJA: Total join arthroplasty
TKA: Total knee arthroplasty
VTE: Venous thromboembolism
