# Supplementary material for: Pharmacological thromboprophylaxis as a risk factor for early periprosthetic joint infection following primary total joint arthroplasty
Source: Sci Rep. 2022 Jun 22;12:10579. doi: 10.1038/s41598-022-14749-y (PMC9217817; doi:10.1038/s41598-022-14749-y)
Supplement: Supplementary file 1 — Supplementary Table S1. [file 41598_2022_14749_MOESM1_ESM.docx]

**Table S1** Univariate and multivariate analysis of factors associated with 30-day readmission for SSC

|  | 30-day readmission for SSC  (n=58) | No 30-day readmission for SSC  (n=7453) | Univariate | | Multivariate | |
| --- | --- | --- | --- | --- | --- | --- |
|  |  |  | P-value | Odds ratio  (95%CI) | P-value | Odds ratio  (95%CI) |
| Age (years) | 67.4±13.1 | 68.7±11.2 | 0.384 | 0.991 (0.969-1.012) |  |  |
| Sex (Male %) | 19 (32.8%) | 1783 (23.9%) | 0.119 | 1.549 (0.893-2.688) |  |  |
| WHO classification of weight status |  |  |  |  |  |  |
| Underweight (%) | 0 (0%) | 102 (1.4%) | 0.997 | 0 |  |  |
| Normal weight (%) | 18 (31%) | 2298 (30.8%) | - | 1 [Reference] | - | 1 [Reference] |
| Pre-obesity (%) | 20 (34.5%) | 3336 (44.8%) | 0.120 | 0.650 (0.377-1.118) |  |  |
| Obesity (%)* | 20 (34.5%) | 1717 (23.0%) | 0.042 | 1.758 (1.020-3.030) | - | - |
| Smoking (%) | 5 (8.6%) | 618 (8.3%) | 0.928 | 1.043 (0.416-2.620) |  |  |
| DM (%) | 21 (63.8%) | 1565 (21.0%) | 0.006 | 2.135 (1.246-3.658) | 0.006 | 2.135 (1.246-3.658) |
| RA (%) | 1 (1.7%) | 195 (2.6%) | 0.673 | 0.653 (0.090-4.740) |  |  |
| Charlson comorbidity index (%) |  |  |  |  |  |  |
| 0 | 5 (8.6%) | 372 (5.0%) | - | 1 [Reference] | - | 1 [Reference] |
| 1 | 4 (6.9%) | 504 (6.8%) | 0.968 | 1.021 (0.368-2.831) |  |  |
| 2 | 7 (12.1%) | 1476 (19.8%) | 0.146 | 0.556 (0.252-1.227) |  |  |
| 3 | 16 (27.6%) | 2255 (30.3%) | 0.659 | 0.878 (0.493-1.565) |  |  |
| 4 | 16 (27.6%) | 1639 (22%) | 0.308 | 1.351 (0.758-2.410) |  |  |
| 5 | 5 (8.6%) | 771 (10.3%) | 0.668 | 0.818 (0.326-2.052) |  |  |
| 6+ | 5 (8.6%) | 436 (5.8%) | 0.375 | 1.518 (0.604-3.818) |  |  |
| History of VTE (%) | 0 (0%) | 195 (2.6%) | 0.999 | 0 |  |  |
| Presence of varicose veins (%) | 2 (3.4%) | 195 (2.6%) | 0.694 | 1.329 (0.322-5.486) |  |  |
| Type of procedure (TKA %) | 45 (77.6%) | 5441 (73.0%) | 0.435 | 1.280 (0.689-2.378) |  |  |
| Bilateral procedure (%) | 14 (24.1%) | 1616 (21.7%) | 0.652 | 1.149 (0.628-2.102) |  |  |
| VTE prophylaxis (%) | 22 (37.9%) | 1935 (26.0%) | 0.041 | 1.743 (1.023-2.969) | - | - |
| Blood transfusion (%) | 24 (41.4%) | 2603 (34.9%) | 0.306 | 1.315 (0.778-2.223) |  |  |

*including obesity class I, II and III
