# Supplementary material for: Pharmacological thromboprophylaxis as a risk factor for early periprosthetic joint infection following primary total joint arthroplasty
Source: Sci Rep. 2022 Jun 22;12:10579. doi: 10.1038/s41598-022-14749-y (PMC9217817; doi:10.1038/s41598-022-14749-y)
Supplement: Supplementary file 2 — Supplementary Table S2. [file 41598_2022_14749_MOESM2_ESM.docx]

**Table S2** Univariate and multivariate analysis of factors associated with 90-day readmission for SSC

|  | 90-day readmission for SSC  (n=71) | No 90-day readmission for SSC  (n=7440) | Univariate | | Multivariate | |
| --- | --- | --- | --- | --- | --- | --- |
|  |  |  | P-value | Odds ratio  (95%CI) | P-value | Odds ratio  (95%CI) |
| Age (years) | 67.7±12.5 | 68.7±11.2 | 0.462 | 0.993 (0.973-1.012) |  |  |
| Sex (Male %) | 22 (31%) | 1780 (23.9%) | 0.168 | 1.428 (0.861-2.368) |  |  |
| WHO classification of weight status |  |  |  |  |  |  |
| Underweight (%) | 0 (0%) | 102 (1.4%) | 0.997 | 0 |  |  |
| Normal weight (%) | 21 (29.6%) | 2295 (30.8%) | - | 1 [Reference] | - | 1 [Reference] |
| Pre-obesity (%) | 25 (35.2%) | 3331 (44.8%) | 0.109 | 0.670 (0.411-1.093) |  |  |
| Obesity (%)* | 25 (35.2%) | 1712 (23.0%) | 0.017 | 1.818 (1.114-2.968) | - | - |
| Smoking (%) | 5 (7.0%) | 618 (8.3%) | 0.701 | 0.836 (0.336-2.083) |  |  |
| DM (%) | 23 (32.4%) | 1563 (21.0%) | 0.021 | 1.802 (1.093-2.971) | 0.046 | 1.637 (1.010-2.773) |
| RA (%) | 3 (4.2%) | 193 (2.6%) | 0.396 | 1.657 (0.517-5.311) |  |  |
| Charlson comorbidity index (%) |  |  |  |  |  |  |
| 0 | 5 (7.0%) | 372 (5.0%) | - | 1 [Reference] | - | 1 [Reference] |
| 1 | 5 (7.0%) | 503 (6.8%) | 0.925 | 1.045 (0.419-2.605) |  |  |
| 2 | 8 (11.3%) | 1475 (19.8%) | 0.077 | 0.514 (0.246-1.074) |  |  |
| 3 | 19 (26.8%) | 2252 (30.3%) | 0.522 | 0.842 (0.497-1.427) |  |  |
| 4 | 22 (31.0%) | 1633 (21.9%) | 0.070 | 1.597 (0.963-2.648) |  |  |
| 5 | 6 (8.5%) | 770 (10.3%) | 0.602 | 0.800 (0.345-1.851) |  |  |
| 6+ | 6 (8.5%) | 435 (5.8%) | 0.356 | 1.486 (0.641-3.450) |  |  |
| History of VTE (%) | 1 (1.4%) | 15 (0.2%) | 0.060 | 7.071 (0.921-54.268) |  |  |
| Presence of varicose veins (%) | 2 (2.8%) | 195 (2.6%) | 0.918 | 1.077 (0.262-4.424) |  |  |
| Type of procedure (TKA %) | 55 (77.5%) | 5431 (73.0%) | 0.400 | 1.272 (0.727-2.224) |  |  |
| Bilateral procedure (%) | 16 (22.5%) | 1614 (21.7%) | 0.865 | 1.050 (0.600-1.837) |  |  |
| VTE prophylaxis (%) | 28 (39.4%) | 1929 (25.9%) | 0.011 | 1.860 (1.153-3.003) | 0.023 | 1.753 (1.081-2.842) |
| Blood transfusion (%) | 29 (40.8%) | 2598 (34.9%) | 0.299 | 1.287 (0.800-2.071) |  |  |

*including obesity class I, II and III
