# Supplementary material for: Pharmacological thromboprophylaxis as a risk factor for early periprosthetic joint infection following primary total joint arthroplasty
Source: Sci Rep. 2022 Jun 22;12:10579. doi: 10.1038/s41598-022-14749-y (PMC9217817; doi:10.1038/s41598-022-14749-y)
Supplement: Supplementary file 3 — Supplementary Table S3. [file 41598_2022_14749_MOESM3_ESM.docx]

**Table S3** Univariate and multivariate analysis of factors associated with all 90-day SSC events

|  | 90-day SSC events  (n=80) | No 90-day SSC events (n=7431) | Univariate | | Multivariate | |
| --- | --- | --- | --- | --- | --- | --- |
|  |  |  | P-value | Odds ratio  (95%CI) | P-value | Odds ratio  (95%CI) |
| Age (years) | 67.3±12.8 | 68.7±11.2 | 0.265 | 0.990 (0.972-1.008) |  |  |
| Sex (Male %) | 25 (31.3%) | 1777 (23.9%) | 0.128 | 1.446 (0.899-2.327) |  |  |
| WHO classification of weight status |  |  |  |  |  |  |
| Underweight (%) | 0 (0%) | 102 (1.4%) | 0.997 | 0 |  |  |
| Normal weight (%) | 24 (30.0%) | 2292 (30.8%) | - | 1 [Reference] | - | 1 [Reference] |
| Pre-obesity (%) | 28 (35.0%) | 3328 (44.8%) | 0.082 | 0.664 (0.418-1.053) |  |  |
| Obesity (%)* | 28 (35.0%) | 1709 (23.0%) | 0.013 | 1.803 (1.135-2.863) | 0.013 | 1.803 (1.135-2.863) |
| Smoking (%) | 6 (7.5%) | 617 (8.3%) | 0.796 | 0.895 (0.388-2.066) |  |  |
| DM (%) | 22 (27.5%) | 1564 (21.0%) | 0.162 | 1.423 (0.868-2.332) |  |  |
| RA (%) | 3 (3.8%) | 193 (2.6%) | 0.522 | 1.461 (0.457-4.671) |  |  |
| Charlson comorbidity index (%) |  |  |  |  |  |  |
| 0 | 7 (8.8%) | 370 (5.0%) | - | 1 [Reference] | - | 1 [Reference] |
| 1 | 6 (7.4%) | 502 (6.8%) | 0.792 | 1.119 (0.485-2.584) |  |  |
| 2 | 9 (11.3%) | 1474 (19.8%) | 0.060 | 0.512 (0.255-1.027) |  |  |
| 3 | 22 (27.5%) | 2249 (30.2%) | 0.592 | 0.874 (0.534-1.431) |  |  |
| 4 | 25 (31.3%) | 1630 (21.9%) | 0.048 | 1.618 (1.005-2.604) | - | - |
| 5 | 6 (7.4%) | 770 (10.4%) | 0.405 | 0.701 (0.304-1.617) |  |  |
| 6+ | 5 (6.3%) | 436 (5.9%) | 0.885 | 1.070 (0.430-2.658) |  |  |
| History of VTE (%) | 1 (1.3%) | 15 (0.2%) | 0.078 | 6.258 (0.817-47.954) |  |  |
| Presence of varicose veins (%) | 2 (2.5%) | 195 (2.6%) | 0.945 | 0.951 (0.232-3.900) |  |  |
| Type of procedure (TKA %) | 59 (73.8%) | 5427 (73.0%) | 0.886 | 1.037 (0.629-1.712) |  |  |
| Bilateral procedure (%) | 17 (21.3%) | 1613 (21.7%) | 0.921 | 0.973 (0.568-1.667) |  |  |
| VTE prophylaxis (%) | 29 (36.3%) | 1928 (25.9%) | 0.039 | 1.623 (1.026-2.568) | - | - |
| Blood transfusion (%) | 32 (40.0%) | 2595 (34.9%) | 0.344 | 1.242 (0.792-1.948) |  |  |

*including obesity class I, II and III
