# Supplementary material for: Pharmacological thromboprophylaxis as a risk factor for early periprosthetic joint infection following primary total joint arthroplasty
Source: Sci Rep. 2022 Jun 22;12:10579. doi: 10.1038/s41598-022-14749-y (PMC9217817; doi:10.1038/s41598-022-14749-y)
Supplement: Supplementary file 4 — Supplementary Table S4. [file 41598_2022_14749_MOESM4_ESM.docx]

**Table S4** Univariate and multivariate analysis of factors associated with 1-year reoperation for SSC

|  | 1-year reoperation for SSC  (n=11) | No 1-year reoperation for SSC  (n=7500) | Univariate | | Multivariate | |
| --- | --- | --- | --- | --- | --- | --- |
|  |  |  | P-value | Odds ratio  (95%CI) | P-value | Odds ratio  (95%CI) |
| Age (years) | 63.5±12.1 | 68.7±11.2 | 0.125 | 0.968 (0.929-1.009) |  |  |
| Sex (Male %) | 4 (36.4%) | 1798 (24.0%) | 0.343 | 1.812 (0.530-6.197) |  |  |
| WHO classification of weight status |  |  |  |  |  |  |
| Underweight (%) | 0 (0%) | 102 (1.4%) | 0.997 | 0 |  |  |
| Normal weight (%) | 2 (18.2%) | 2314(30.8%) | - | 1 [Reference] | - | 1 [Reference] |
| Pre-obesity (%) | 5 (45.4%) | 3351 (44.7%) | 0.959 | 1.032 (0.315-3.384) |  |  |
| Obesity (%)* | 4 (36.4%) | 1733 (23.1%) | 0.306 | 1.902 (0.556-6.503) |  |  |
| Smoking (%) | 2 (18.2%) | 621 (8.3%) | 0.250 | 2.462 (0.531-11.418) |  |  |
| DM (%) | 2 (18.2%) | 1584 (21.1%) | 0.812 | 0.830 (0.179-3.845) |  |  |
| RA (%) | 0 (0%) | 196 (2.6%) | 0.996 | 0 |  |  |
| Charlson comorbidity index (%) |  |  |  |  |  |  |
| 0 | 0 (0%) | 377 (5.0%) | - | 1 [Reference] | - | 1 [Reference] |
| 1 | 3 (27.3%) | 505 (6.7%) | 0.015 | 5.194 (1.374-19.639) | - | - |
| 2 | 3 (27.3%) | 1480 (19.7%) | 0.533 | 1.525 (0.404-5.757) |  |  |
| 3 | 3 (27.3%) | 2268 (30.3%) | 0.831 | 0.865 (0.229-3.264) |  |  |
| 4 | 0 (0%) | 1655 (22.1%) | 0.988 | 0.000 (0.000-) |  |  |
| 5 | 2 (18.1%) | 774 (10.3%) | 0.400 | 1.931 (0.416-8.954) |  |  |
| 6+ | 0 (0%) | 441 (5.9%) | 0.994 | 0 |  |  |
| History of VTE (%) | 0 (0%) | 16 (0.2%) | 0.999 | 0 |  |  |
| Presence of varicose veins (%) | 1 (9.1%) | 196 (2.6%) | 0.211 | 3.727 (0.475-29.253) |  |  |
| Type of procedure (TKA %) | 9 (81.8%) | 5477 (73.0%) | 0.516 | 1.662 (0.359-7.699) |  |  |
| Bilateral procedure (%) | 2 (18.2%) | 1628 (21.7%) | 0.777 | 0.801 (0.173-3.713) |  |  |
| VTE prophylaxis (%) | 4 (36.4%) | 1953 (26.0%) | 0.440 | 1.623 (0.475-5.550) |  |  |
| Blood transfusion (%) | 4 (36.4%) | 2623 (35.0%) | 0.923 | 1.062 (0.311-3.633) |  |  |

*including obesity class I, II and III
