# Supplementary material for: Pharmacological thromboprophylaxis as a risk factor for early periprosthetic joint infection following primary total joint arthroplasty
Source: Sci Rep. 2022 Jun 22;12:10579. doi: 10.1038/s41598-022-14749-y (PMC9217817; doi:10.1038/s41598-022-14749-y)
Supplement: Supplementary file 5 — Supplementary Table S5. [file 41598_2022_14749_MOESM5_ESM.docx]

**Table S5** Univariate and multivariate analysis of factors associated with 30-day readmission for PJI

|  | 30-day readmission for PJI  (n=9) | No 30-day readmission for PJI  (n=7502) | Univariate | | Multivariate | |
| --- | --- | --- | --- | --- | --- | --- |
|  |  |  | P-value | Odds ratio  (95%CI) | P-value | Odds ratio  (95%CI) |
| Age (years) | 64.3±11.8 | 68.7±11.2 | 0.247 | 0.972 (0.829-1.020) |  |  |
| Sex (Male %) | 3 (33.3%) | 1799 (24.0%) | 0.515 | 1.585 (0.396-6.344) |  |  |
| WHO classification of weight status |  |  |  |  |  |  |
| Underweight (%) | 1 (11.1%) | 101 (1.3%) | 0.038 | 9.160 (1.135-73.915) | 0.022 | 11.692 (1.417-96.482) |
| Normal weight (%) | 4 (44.5%) | 2312 (30.8%) | - | 1 [Reference] | - | 1 [Reference] |
| Pre-obesity (%) | 3 (33.3%) | 3353 (44.7%) | 0.497 | 0.619 (0.155-2.476) |  |  |
| Obesity (%)* | 1 (11.1%) | 1736 (23.2%) | 0.407 | 0.415 (0.052-3.322) |  |  |
| Smoking (%) | 0 (0%) | 623 (8.3%) | 0.993 | 0 |  |  |
| DM (%) | 3 (33.3%) | 1583 (21.1%) | 0.377 | 1.870 (0.467-7.484) |  |  |
| RA (%) | 1 (11.1%) | 195 (2.6%) | 0.146 | 4.684 (0.583-37.632) |  |  |
| Charlson comorbidity index (%) |  |  |  |  |  |  |
| 0 | 0 (0%) | 377 (5.0%) | - | 1 [Reference] | - | 1 [Reference] |
| 1 | 1 (11.1%) | 507 (6.8%) | 0.608 | 1.725 (0.215-13.816) |  |  |
| 2 | 3 (33.4%) | 1480 (19.7%) | 0.316 | 2.034 (0.508-8.144) |  |  |
| 3 | 3 (33.4%) | 2268 (30.2%) | 0.840 | 1.154 (0.288-4.618) |  |  |
| 4 | 1 (11.1%) | 1654 (22.1%) | 0.442 | 0.442 (0.055-3.536) |  |  |
| 5 | 1 (11.1%) | 775 (10.3%) | 0.939 | 1.085 (0.136-8.686) |  |  |
| 6+ | 0 (0%) | 441 (5.9%) | 0.994 | 0 |  |  |
| History of VTE (%) | 0 (0%) | 16 (0.2%) | 0.999 | 0 |  |  |
| Presence of varicose veins (%) | 0 (0%) | 197 (2.6%) | 0.996 | 0 |  |  |
| Type of procedure (TKA %) | 6 (66.7%) | 5480 (73.1%) | 0.688 | 0.738 (0.184-2.953) |  |  |
| Bilateral procedure (%) | 5 (55.6%) | 1625 (21.7%) | 0.025 | 4.520 (1.212-16.852) | 0.019 | 4.882 (1.295-18.400) |
| VTE prophylaxis (%) | 4 (44.4%) | 1953 (26.0%) | 0.221 | 2.273 (0.610-8.473) |  |  |
| Blood transfusion (%) | 5 (55.6%) | 2622 (35.0%) | 0.208 | 2.326 (0.624-8.671) |  |  |

*including obesity class I, II and III
