# Supplementary material for: Pharmacological thromboprophylaxis as a risk factor for early periprosthetic joint infection following primary total joint arthroplasty
Source: Sci Rep. 2022 Jun 22;12:10579. doi: 10.1038/s41598-022-14749-y (PMC9217817; doi:10.1038/s41598-022-14749-y)
Supplement: Supplementary file 6 — Supplementary Table S6. [file 41598_2022_14749_MOESM6_ESM.docx]

**Table S6** Univariate and multivariate analysis of factors associated with 90-day readmission for PJI

|  | 90-day readmission for PJI  (n=12) | No 90-day readmission for PJI  (n=7499) | Univariate | | Multivariate | |
| --- | --- | --- | --- | --- | --- | --- |
|  |  |  | P-value | Odds ratio  (95%CI) | P-value | Odds ratio  (95%CI) |
| Age (years) | 62.9±12.3 | 68.7±11.2 | 0.078 | 0.966 (0.929-1.004) | 0.046 | 0.959 (0.920-0.999) |
| Sex (Male %) | 5 (41.7%) | 1797 (24.0%) | 0.163 | 2.266 (0.718-7.150) |  |  |
| WHO classification of weight status |  |  |  |  |  |  |
| Underweight (%) | 1 (8.3%) | 101 (1.4%) | 0.071 | 6.659 (0.852-52.063) |  |  |
| Normal weight (%) | 5 (41.7%) | 2311 (30.8%) | - | 1 [Reference] | - | 1 [Reference] |
| Pre-obesity (%) | 3 (25.0%) | 3353 (44.7%) | 0.184 | 0.412 (0.111-1.524) |  |  |
| Obesity (%)* | 3 (25.0%) | 1734 (23.1%) | 0.878 | 1.108 (0.300-4.098) |  |  |
| Smoking (%) | 0 (0%) | 623 (8.3%) | 0.993 | 0 |  |  |
| DM (%) | 3 (25.0%) | 1583 (21.1%) | 0.742 | 1.246 (0.337-4.607) |  |  |
| RA (%) | 1 (8.3%) | 195 (2.6%) | 0.242 | 3.405 (0.437-26.505) |  |  |
| Charlson comorbidity index (%) |  |  |  |  |  |  |
| 0 | 1 (8.3%) | 376 (5.0%) | - | 1 [Reference] | - | 1 [Reference] |
| 1 | 1 (8.3%) | 507 (6.8%) | 0.829 | 1.254 (0.162-9.730) |  |  |
| 2 | 3 (25.0%) | 1480 (19.7%) | 0.648 | 1.356 (0.367-5.014) |  |  |
| 3 | 4 (33.4%) | 2267 (30.2%) | 0.815 | 1.154 (0.347-3.836) |  |  |
| 4 | 2 (16.7%) | 1653 (22.0%) | 0.655 | 0.707 (0.155-3.231) |  |  |
| 5 | 1 (8.3%) | 775 (10.3%) | 0.820 | 0.789 (0.102-6.117) |  |  |
| 6+ | 0 (0%) | 441 (5.9%) | 0.994 | 0 |  |  |
| History of VTE (%) | 0 (0%) | 16 (0.2%) | 0.999 | 0 |  |  |
| Presence of varicose veins (%) | 0 (0%) | 197 (2.6%) | 0.996 | 0 |  |  |
| Type of procedure (TKA %) | 9 (75.0%) | 5477 (73.0%) | 0.878 | 1.108 (0.300-4.095) |  |  |
| Bilateral procedure (%) | 5 (41.7%) | 1625 (21.7%) | 0.106 | 2.582 (0.818-8.144) |  |  |
| VTE prophylaxis (%) | 6 (50.0%) | 1951 (26.0%) | 0.071 | 2.844 (0.916-8.827) | 0.045 | 3.267 (1.026-10.402) |
| Blood transfusion (%) | 6 (50.0%) | 2621 (35.0%) | 0.282 | 1.861 (0.600-5.776) |  |  |

*including obesity class I, II and III
