# Supplementary material for: Pharmacological thromboprophylaxis as a risk factor for early periprosthetic joint infection following primary total joint arthroplasty
Source: Sci Rep. 2022 Jun 22;12:10579. doi: 10.1038/s41598-022-14749-y (PMC9217817; doi:10.1038/s41598-022-14749-y)
Supplement: Supplementary file 7 — Supplementary Table S7. [file 41598_2022_14749_MOESM7_ESM.docx]

**Table S7** Univariate and multivariate analysis of factors associated with all 90-day PJI events

|  | 90-day  PJI events  (n=16) | No 90-day  PJI events (n=7495) | Univariate | | Multivariate | |
| --- | --- | --- | --- | --- | --- | --- |
|  |  |  | P-value | Odds ratio  (95%CI) | P-value | Odds ratio  (95%CI) |
| Age (years) | 63.9±14.1 | 68.7±11.2 | 0.092 | 0.970 (0.937-1.005) |  |  |
| Sex (Male %) | 8 (50.0%) | 1794 (24.0%) | 0.021 | 3.178 (1.191-8.479) | 0.012 | 3.568 (1.328-9.583) |
| WHO classification of weight status |  |  |  |  |  |  |
| Underweight (%) | 1 (6.3%) | 101 (1.4%) | 0.127 | 4.881 (0.639-37.300) |  |  |
| Normal weight (%) | 6 (37.5%) | 2310 (30.8%) | - | 1 [Reference] | - | 1 [Reference] |
| Pre-obesity (%) | 5 (31.2%) | 3351 (44.7%) | 0.286 | 0.562 (0.195-1.619) |  |  |
| Obesity (%)* | 4 (25.0%) | 1733 (23.1%) | 0.859 | 1.108 (0.357-3.441) |  |  |
| Smoking (%) | 1 (6.3%) | 622 (8.3%) | 0.767 | 0.737 (0.097-5.586) |  |  |
| DM (%) | 6 (37.5%) | 1580 (21.1%) | 0.118 | 2.246 (0.815-6.190) |  |  |
| RA (%) | 1 (6.3%) | 195 (2.6%) | 0.377 | 2.496 (0.328-18.988) |  |  |
| Charlson comorbidity index (%) |  |  |  |  |  |  |
| 0 | 2 (12.5%) | 375 (5.0%) | - | 1 [Reference] | - | 1 [Reference] |
| 1 | 1 (6.3%) | 507 (6.8%) | 0.935 | 0.919 (0.121-6.970) |  |  |
| 2 | 3 (18.7%) | 1480 (19.7%) | 0.920 | 0.938 (0.267-3.295) |  |  |
| 3 | 5 (31.2%) | 2266 (30.2%) | 0.930 | 1.049 (0.364-3.022) |  |  |
| 4 | 2 (12.5%) | 1653 (22.1%) | 0.366 | 0.505 (0.115-2.224) |  |  |
| 5 | 1 (6.3%) | 775 (10.3%) | 0.596 | 0.578 (0.076-4.382) |  |  |
| 6+ | 2 (12.5%) | 439 (5.9%) | 0.273 | 2.296 (0.520-10.135) |  |  |
| History of VTE (%) | 0 (0%) | 16 (0.2%) | 0.999 | 0 |  |  |
| Presence of varicose veins (%) | 1 (6.3%) | 196 (2.6%) | 0.380 | 2.483 (0.326-18.888) |  |  |
| Type of procedure (TKA %) | 11 (68.8%) | 5475 (73.1%) | 0.699 | 0.812 (0.282-2.339) |  |  |
| Bilateral procedure (%) | 6 (37.5%) | 1624 (21.7%) | 0.134 | 2.169 (0.787-5.976) |  |  |
| VTE prophylaxis (%) | 8 (50.0%) | 1949 (26.0%) | 0.037 | 2.846 (1.067-7.592) | 0.020 | 3.222 (1.200-8.656) |
| Blood transfusion (%) | 8 (50.0%) | 2619 (34.9%) | 0.214 | 1.862 (0.698-4.966) |  |  |

*including obesity class I, II and III
