# Supplementary material for: Pharmacological thromboprophylaxis as a risk factor for early periprosthetic joint infection following primary total joint arthroplasty
Source: Sci Rep. 2022 Jun 22;12:10579. doi: 10.1038/s41598-022-14749-y (PMC9217817; doi:10.1038/s41598-022-14749-y)
Supplement: Supplementary file 8 — Supplementary Table S8. [file 41598_2022_14749_MOESM8_ESM.docx]

**Table S8** Univariate and multivariate analysis of factors associated with 1-year reoperation for PJI

|  | 1-year reoperation  for PJI  (n=29) | No 1-year reoperation  for PJI (n=7482) | Univariate | | Multivariate | |
| --- | --- | --- | --- | --- | --- | --- |
|  |  |  | P-value | Odds ratio  (95%CI) | P-value | Odds ratio  (95%CI) |
| Age (years) | 67.3±11.1 | 68.7±11.2 | 0.496 | 0.990 (0.960-1.020) |  |  |
| Sex (Male %) | 13 (44.8%) | 1789 (23.9%) | 0.011 | 2.586 (1.241-5.385) | 0.008 | 2.738 (1.308-5.731) |
| WHO classification of weight status |  |  |  |  |  |  |
| Underweight (%) | 2 (6.9%) | 100 (1.3%) | 0.022 | 5.468 (1.283-23.307) | - | - |
| Normal weight (%) | 9 (31.0%) | 2307 (30.83%) | - | 1 [Reference] | - | 1 [Reference] |
| Pre-obesity (%) | 13 (44.8%) | 3343 (44.7%) | 0.987 | 1.006 (0.483-2.094) |  |  |
| Obesity (%)* | 5 (17.2%) | 1732 (23.2%) | 0.454 | 0.692 (0.264-1.815) |  |  |
| Smoking (%) | 3 (10.3%) | 620 (8.3%) | 0.689 | 1.277 (0.385-4.231) |  |  |
| DM (%) | 6 (20.7%) | 1580 (21.1%) | 0.955 | 0.974 (0.396-2.397) |  |  |
| RA (%) | 3 (10.3%) | 193 (2.6%) | 0.017 | 4.358 (1.308-14.520) | 0.009 | 5.048 (1.501-16.974) |
| Charlson comorbidity index (%) |  |  |  |  |  |  |
| 0 | 1 (3.5%) | 376 (5.0%) | - | 1 [Reference] | - | 1 [Reference] |
| 1 | 3 (10.3%) | 505 (6.7%) | 0.446 | 1.594 (0.481-5.285) |  |  |
| 2 | 5 (17.2%) | 1478 (19.8%) | 0.735 | 0.846 (0.322-2.222) |  |  |
| 3 | 11 (38.0%) | 2260 (30.2%) | 0.368 | 1.412 (0.666-2.994) |  |  |
| 4 | 5 (17.2%) | 1650 (22.1%) | 0.534 | 0.736 (0.281-1.933) |  |  |
| 5 | 2 (6.9%) | 774 (10.3%) | 0.546 | 0.642 (0.152-2.705) |  |  |
| 6+ | 2 (6.9%) | 439 (5.9%) | 0.814 | 1.188 (0.282-5.014) |  |  |
| History of VTE (%) | 0 (0%) | 16 (0.2%) | 0.999 | 0 |  |  |
| Presence of varicose veins (%) | 1 (3.5%) | 196 (2.6%) | 0.781 | 1.328 (0.180-9.807) |  |  |
| Type of procedure (TKA %) | 21 (72.4%) | 5465 (73.0%) | 0.939 | 0.969 (0.428-2.191) |  |  |
| Bilateral procedure (%) | 6 (20.7%) | 1624 (21.7%) | 0.894 | 0.941 (0.382-2.314) |  |  |
| VTE prophylaxis (%) | 8 (27.6%) | 1949 (26.1%) | 0.851 | 1.081 (0.478-2.446) |  |  |
| Blood transfusion (%) | 12 (41.4%) | 2615 (35.0%) | 0.470 | 1.314 (0.627-2.755) |  |  |

*including obesity class I, II and III
